# Unusually high soil nitrogen oxide emissions influence air quality in a high-temperature agricultural region

**DOI:** 10.1038/ncomms9753

**Published:** 2015-11-10

**Authors:** P. Y. Oikawa, C. Ge, J. Wang, J. R. Eberwein, L. L. Liang, L. A. Allsman, D. A. Grantz, G. D. Jenerette

**Affiliations:** 1 Department of Environmental Science, Policy and Management, University of California, Berkeley, California 94720, USA; 2 Department of Earth and Atmospheric Sciences, University of Nebraska-Lincoln, Lincoln, Nebraska 68588, USA; 3 Department of Botany and Plant Sciences, University of California, Riverside, California 92521, USA

## Abstract

Fertilized soils have large potential for production of soil nitrogen oxide (NO_x_=NO+NO_2_), however these emissions are difficult to predict in high-temperature environments. Understanding these emissions may improve air quality modelling as NO_x_ contributes to formation of tropospheric ozone (O_3_), a powerful air pollutant. Here we identify the environmental and management factors that regulate soil NO_x_ emissions in a high-temperature agricultural region of California. We also investigate whether soil NO_x_ emissions are capable of influencing regional air quality. We report some of the highest soil NO_x_ emissions ever observed. Emissions vary nonlinearly with fertilization, temperature and soil moisture. We find that a regional air chemistry model often underestimates soil NO_x_ emissions and NO_x_ at the surface and in the troposphere. Adjusting the model to match NO_x_ observations leads to elevated tropospheric O_3_. Our results suggest management can greatly reduce soil NO_x_ emissions, thereby improving air quality.

## Abstract (web summary)

Soil NO_x_ emissions can significantly impact air quality in agricultural regions, particularly high temperature fertilized systems. Here, the authors investigate NO_x_ emissions in one such system in California and suggest that the NO_x_ emissions are the highest ever observed, with implications for air quality.

## Graphical abstract

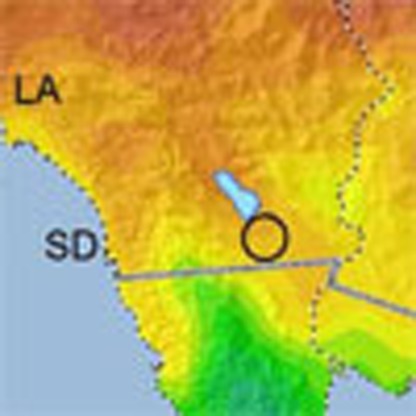

While agriculture in high-temperature environments is widespread and will become increasingly prominent in a future warmer climate^1^, the impacts of these systems on air quality are poorly constrained^2^,^3^. Nitrogen (N) losses to the atmosphere from high-temperature agroecosystems are not well characterized^2^,^4^ and are likely higher than temperate systems due to the combination of N fertilization^2^,^5^, nonlinear temperature dependence of biological processes^6^ and pulsed fluxes in response to irrigation—drying cycles^7^. Soil nitrogen oxide (NO_x_=NO+NO_2_) is one important form of N trace gas that can be released from fertilized soils and plays an important role in the formation of tropospheric ozone (O_3_), a toxic air pollutant. Approximately 1/4 of global NO_x_ production is derived from soils, mostly from fertilized agriculture; however, estimates of global soil NO_x_ emissions vary widely (9–27 Tg per year)^8^,^9^,^10^. Understanding how soil NO_x_ emissions are regulated in high-temperature agroecosystems will help constrain current and future global NO_x_ budgets and quantify the human health and ecosystem impacts of fertilized agriculture in a warming world.

The Southwestern United States of America has been experiencing warmer winter temperatures and more frequent heat waves over the past 100 years^11^,^12^ and is considered to be a climate-change hotspot^13^. The Imperial Valley, CA, is an important agricultural region within the Southwestern United States of America encompassing 200,000 hectares of irrigated agricultural land with air temperatures >40 °C in the summer. The Imperial Valley also suffers from poor air quality that regularly exceeds government O_3_ standards^14^, and experiences the highest rates of asthma hospitalizations in California^15^. To improve air quality in the region, understanding how urban and agricultural sources contribute to O_3_ formation is necessary. Fossil fuel combustion is likely a dominant source of NO_x_ in the region, as there are small cities within the Imperial Valley (for example, El Centro; population=163,972) and large neighbouring urban areas including Los Angeles, San Diego and Mexicali. However, it is not clear whether agricultural NO_x_ emissions significantly increase O_3_ formation, as O_3_ chemistry may be NO_x_ saturated^16^. On the other hand, if the atmosphere is NO_x_ limited, soil NO_x_ emissions may enhance O_3_ formation, as observed in agricultural regions in the Midwestern United States of America^17^. The Imperial Valley is therefore a complex and important location for studying the impact of agriculture on air quality and human health.

Soil NO_x_ emissions vary nonlinearly with environmental and land management factors including temperature, fertilization and soil moisture, but these relationships are not well constrained in high-temperature systems. While most studies have detected exponential increases in soil NO_x_ emissions with temperature, there are contrasting results concerning high-temperature (>30 °C) responses of soil NO_x_ emission^18^,^19^. Fertilization and N deposition are known to increase soil NO_x_ emissions; however, the majority of studies are conducted at temperatures below 35 °C (refs 6, 20). In addition, fertilization type, amount and application method are known to influence soil NO_x_ emissions. Side-injected fertilizers (where fertilizer is injected into the soil versus applied to the top) and splitting fertilization into smaller applications (<100 kg N ha^−1^) can limit NO_x_ emission; however, these factors have mainly been evaluated in temperate environments^5^. Finally, irrigation and soil moisture are important factors regulating soil NO_x_ emissions. In particular, strong pulse NO_x_ emission responses to rewetting of soils in high-temperature regions are important^21^,^22^,^23^, yet understudied in managed systems. Therefore, measuring soil NO_x_ emissions at high temperatures under different fertilization and soil-moisture conditions is needed to understand the regulation of fluxes, improve management and inform biogeochemical models.

Most chemistry transport models predict soil NO_x_ emissions as a function of temperature, soil moisture and ecosystem type, such as in the Yienger and Levy model^24^ (hereafter called YL95). Models often assume optimum temperatures for nitrification and denitrification occur at 20–30 °C (refs 25, 26), with soil NO_x_ emissions increasing exponentially before hitting a plateau at 30 °C (refs 8, 9). Within the YL95 paradigm, agricultural systems are assumed to be wet year-round and are assigned an emission factor associated with fertilization, where 1–2% of fertilizer applied is lost as NO_x_ throughout the growing season^27^,^28^. Fertilization and irrigation events are generally not considered pulse events in models and are instead interpreted as elevating emissions at a constant rate throughout a growing season (for example, YL95 and the Weather Research and Forecasting with Chemistry model (WRF-Chem)^29^). Despite updates to YL95 (refs 8, 9), uncertainty associated with NO_x_ pulse emission events and fertilizer-induced agricultural emissions is large^8^,^9^,^23^,^27^.

Here we aim to identify the environmental (that is, soil temperature, soil volumetric water content and inorganic N availability) and management factors (that is, time since fertilization and fertilization application practices) that regulate soil NO_x_ emission, and its impacts on air quality in a high-temperature agricultural region: the Imperial Valley, California. Soil NO_x_ emission measurements were conducted using the static chamber technique throughout two growing seasons of a high biomass grass, *Sorghum bicolor*. We use two N-fertilization experiments to evaluate pulse NO_x_ responses to different levels of fertilization (20, 50 and 100 kg N ha^−1^) and fertilizer application methods (side-injected dry granulated N versus dissolved N). The implications of soil NO_x_ fluxes on regional air quality are evaluated using a regional air chemistry model and local and remote sensing measurements of NO_2_ at the surface and in the troposphere. In these modelling exercises, we aim to test whether the model assumptions are valid for high-temperature environments. Specifically, we evaluate the model's ability to simulate soil NO_x_ emissions, surface concentrations of NO_2_ and tropospheric NO_2_ columns in the Imperial Valley. We also elevate soil NO_x_ emission rates in the model to evaluate the impact of those emissions on regional concentrations of tropospheric O_3_. We find that soil NO_x_ emissions from this site are among the highest ever observed and respond nonlinearly to increases in fertilization, temperature and soil moisture. We also find that a regional air chemistry model often underestimates soil NO_x_ emissions, surface NO_2_ concentrations and tropospheric NO_2_ columns in the Imperial Valley. Finally, we find that increasing cropland soil NO_x_ emissions to match observations leads to elevated surface O_3_ concentrations.

## Results

### Environmental drivers of soil NO_x_ emission

**Figure 1:**
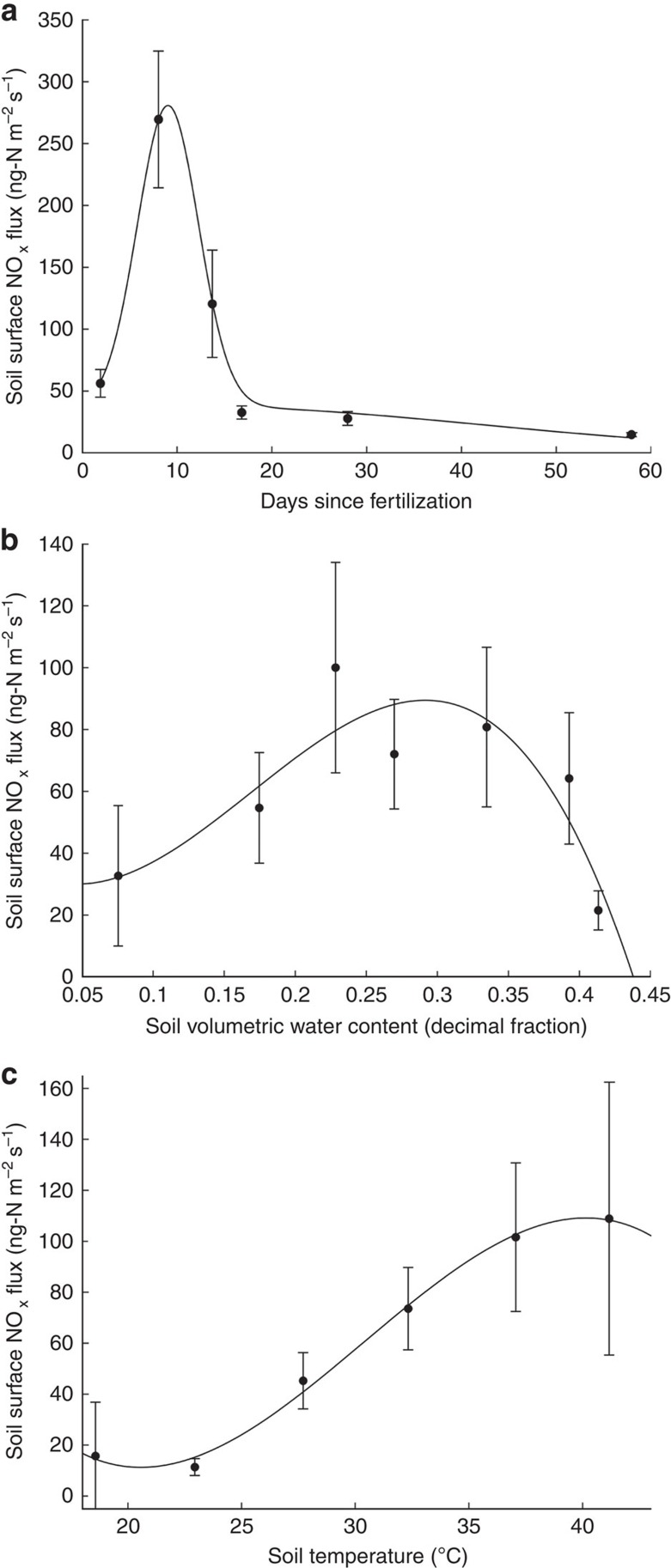
Environmental variables that regulate soil NO_x_ emission. Relationship between soil surface NO_x_ flux (ng N m^−2^ s^−1^) and (**a**) days since fertilization, (**b**) soil volumetric water content (0–10 cm) and (**c**) soil temperature (average 2 and 10 cm; °C). Measurements were collected across two growing seasons 2012–2013, including measurements made during fertilization experiments (*n*=241). Data were binned by environmental variable and s.e. bars are shown for each bin. A Gaussian equation (*f*(*x*)=a+b*exp(−0.5((*x*−*c*)/*d*)^2^)) was fitted to the NO_x_ relationship with days since fertilization data (*a*=18.6, *b*=254.3, *c*=8.6 and *d*=3.4). A third-order polynomial (*f*(*x*)=a*x^3^+b*x^2^+c*x+*d*) was fitted to the NO_x_ relationship with soil volumetric water content (*a*=−8128.3, *b*=4125.5, *c*=−333.3 and *d*=37.4) and soil temperature (*a*=−0.026, *b*=2.4, *c*=−64.6 and *d*=562.5).

Soil NO_x_ emissions observed in a high-temperature fertilized agricultural region of the Imperial Valley, CA, ranged between −5 and 900 ng N m^−2^ s^−1^. The highest NO_x_ fluxes (top 10%) were observed at temperatures between 27–40 °C with moderate soil volumetric water content (0.14–0.40) and within 23 days of a fertilization event. The strongest predictor of NO_x_ flux across all measurements was days since fertilization (*F*=5.32, *P*<0.0001; Fig. 1a) followed by soil volumetric water content (averaged across 0–10 cm depth; *F*=3.55, *P*=0.00053; Fig. 1b) and soil temperature (averaged between 2 and 10 cm depth; *F*=2.69, *P*=0.016; Fig. 1c). The adjusted *R*^2^ for the nonlinear model was 0.38, with 48.2% of deviance explained. Inorganic soil N content (NH_4_ and NO_3_) was a weak predictor of flux (NH_4_: *F*=3.04, *P*=0.043; NO_3_: *F*=2.28, *P*=0.061).

### Soil NO_x_ emission responses to fertilization and irrigation

**Figure 2:**
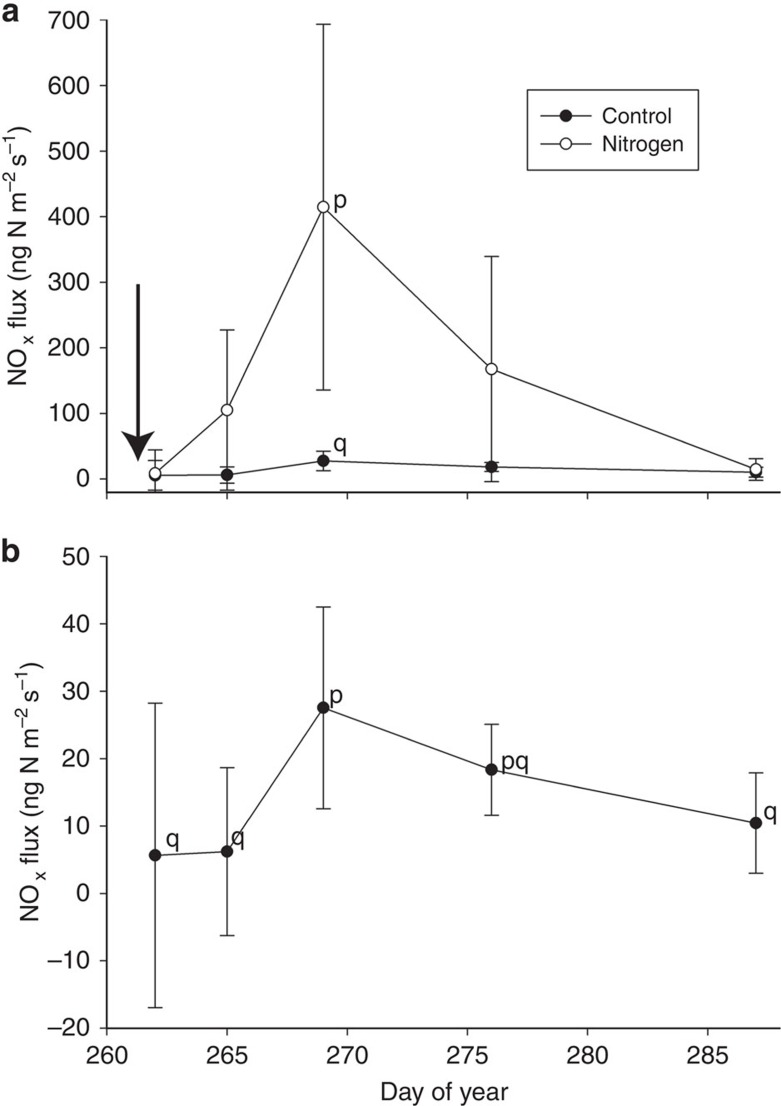
Pulse NO_x_ emission responses to dissolved ammonium nitrate fertilization. (**a**) Soil NO_x_ fluxes observed during a N-fertilization experiment in 2012 where collars received 20 kg ammonium nitrate-N ha^−1^ in dissolved form during an irrigation (white circles; *n*=10) and control collars received irrigation only (black circles; *n*=10; s.d. bars shown). The irrigation and fertilization event is indicated by an arrow (DOY 262). N treatment significantly increased NO_x_ emissions with peak emissions observed 7 days post fertilization. (**b**) Control collar NO_x_ fluxes were also evaluated in a separate analysis to investigate the influence of irrigation alone on NO_x_ flux. Control NO_x_ fluxes were significantly affected by time during the experiment in response to irrigation, where fluxes observed during the third measurement (7 days post treatment) were significantly different from all other measurements except for the fourth measurement (14 days post treatment). Significant differences between time points are indicated by letters (p, q) at *P*<0.05, as determined using pairwise comparisons with Bonferroni adjustment.

In the first N-fertilization experiment, experimental collars received an irrigation and fertilizer treatment (20 kg N ha^−1^ dissolved ammonium nitrate) and control collars received irrigation only. During the experiment, soil temperatures at 2 cm depth were on average 30.0 °C (s.d.=4.5 °C), and plant canopy height was on average 152.3 cm (15.8 s.d.). N-fertilization treatment and time had a significant effect on NO_x_ emissions (*F*=30.56, *P*<0.0001; *F*=12.13, *P*<0.0001 for treatment and time effects, respectively). Pairwise comparisons indicated that the fertilized collars were significantly different from control collars 7 days post treatment (Fig. 2a; *P*<0.01); all other time points were not significantly different from each other. Numerical integration via the trapezoidal method revealed that treatment collars released 0.13 g N-NO_x_ m^−2^ on average during the experiment, corresponding to a 6.6% emission factor. Control collars released 0.012 g N-NO_x_ m^−2^ during the experiment.

To evaluate the importance of rewetting events for NO_x_ emissions without a recent fertilization, we conducted a separate analysis of the above experiment, but only included control collars. These control collars received irrigation only and had not been fertilized for over 30 days. In this separate analysis, NO_x_ fluxes from control collars were significantly affected by time in response to irrigation (Fig. 2b; *F*=4.01, *P*<0.01), where the third measurement (7 days post treatment) was significantly different from all previous measurements (*P*<0.05), but not the fourth measurement (14 days post treatment; Fig. 2b), indicating that pulse soil NO_x_ emission responses to irrigation occur even over 30 days after fertilization.

**Figure 3:**
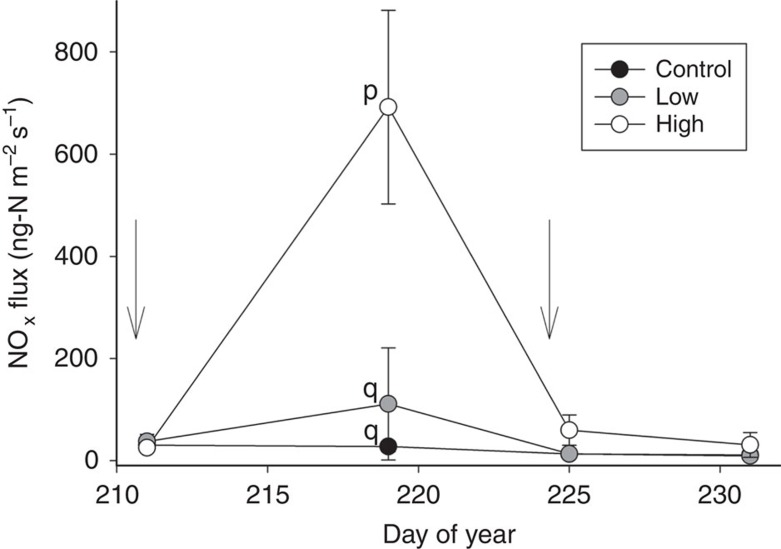
Pulse NO_x_ emission responses to side-injected urea fertilization. Soil NO_x_ fluxes observed during a fertilization experiment in 2013 with control (0 N), low (50 kg urea-N ha^−1^) and high (100 kg urea-N ha^−1^) side-injected fertilization treatments (*n*=3 collars per treatment; s.d. bars shown). Irrigation events are indicated by arrows. Fertilization occurred on DOY 210. High-fertilizer treatment collars were significantly different from low-fertilizer treatment and control collars at the second time point (9 days post treatment); no differences between treatments were detected at other time points. Significant differences between time points are indicated by letters (p, q) at *P*<0.05, as determined using pairwise comparisons with Bonferroni adjustment.

In the second N-fertilization experiment, experimental collars received low (50 kg N ha^−1^) or high (100 kg N ha^−1^) fertilizer treatment plus irrigation, while control collars received irrigation only. Fertilizer was applied via a side injection with urea granules. During the experiment, soil temperatures at 2 cm depth were on average 37.7 °C (s.d.=4.2 °C), and plant canopy height was on average 57.8 cm (22.7 s.d.). N-fertilization treatment and time had a significant effect on NO_x_ emissions (*F*=32.20, *P*<0.0001, *F*=36.88, *P*<0.0001 for treatment and time effects, respectively). Pairwise comparisons indicated that the high-fertilizer treatment collars were significantly different from low-fertilizer treatment and control collars 9 days post treatment (Fig. 3; *P*<0.05); no differences between treatments were detected at other time points. Numerical integration via the trapezoidal method revealed that while control collars released 0.037 g N-NO_x_ m^−2^ during the experiment, high-fertilizer treatment collars released 0.46 g N-NO_x_ m^−2^ on average during the experiment, corresponding to a 4.6% emission factor, and low-fertilizer treatment collars released 0.089 g N-NO_x_ m^−2^, corresponding to a 1.8% emission factor. Therefore, doubling the fertilization amount (50–100 kg N ha^−1^) increased integrated fluxes by a factor of 5.

### Investigating the regional significance of soil NO_x_ emission

To assess the influence of soil NO_x_ emissions on regional air quality, we employed a regional air chemistry model, WRF-Chem, and local and remotely sensed measurements of NO_2_ in the troposphere. We then increased soil NO_x_ emission rates within the model to reach levels similar to those measured in the field and compared the modelled NO_2_ (default and elevated simulations) with measured surface NO_2_ concentrations and tropospheric NO_2_ columns. Finally, we evaluated the effect of increasing soil NO_x_ emissions on modelled tropospheric O_3_.

First, we compared soil NO_x_ emissions modelled in WRF-Chem with emissions measured in the field. By default, WRF-Chem estimated surface NO_x_ emissions in the Imperial Valley to be near 2 ng NO_x_-N m^−2^ s^−1^. Across all our measurements, NO_x_ fluxes were on average 66.4 ng NO_x_-N m^−2^ s^−1^ with a median of 20 ng NO_x_-N m^−2^ s^−1^. Across measurements made within 20 days of a fertilization event, NO_x_ fluxes were on average 128.1 ng NO_x_-N m^−2^ s^−1^ with a median of 38 ng NO_x_-N m^−2^ s^−1^. By multiplying WRF-Chem emission rates by factors of 10 and 64.5, we elevated soil NO_x_ emissions in Imperial Valley croplands to be near 20 and 129 ng NO_x_-N m^−2^ s^−1^, which are representative of the range in mean and median flux values collected under both average and recently fertilized conditions in the field.

**Figure 4:**
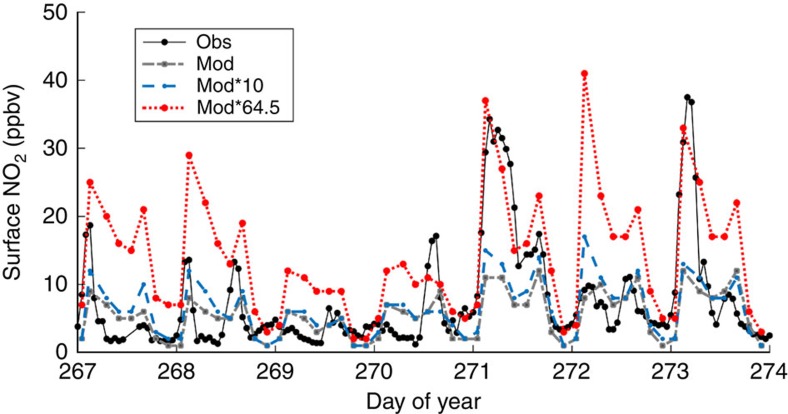
Comparing time series of modelled and measured surface concentrations of NO_2_ in the Imperial Valley, CA. Surface concentrations of NO_2_ measured at an air quality monitoring station (obs) in El Centro, CA (CA Air Resources Board) and modelled with WRF-Chem (mod) and WRF-Chem with soil NO_2_ emission rates multiplied by 10 (mod*10) and 64.5 × (mod*64.5). All data are from 23 to 29 September 2012.

**Figure 5:**
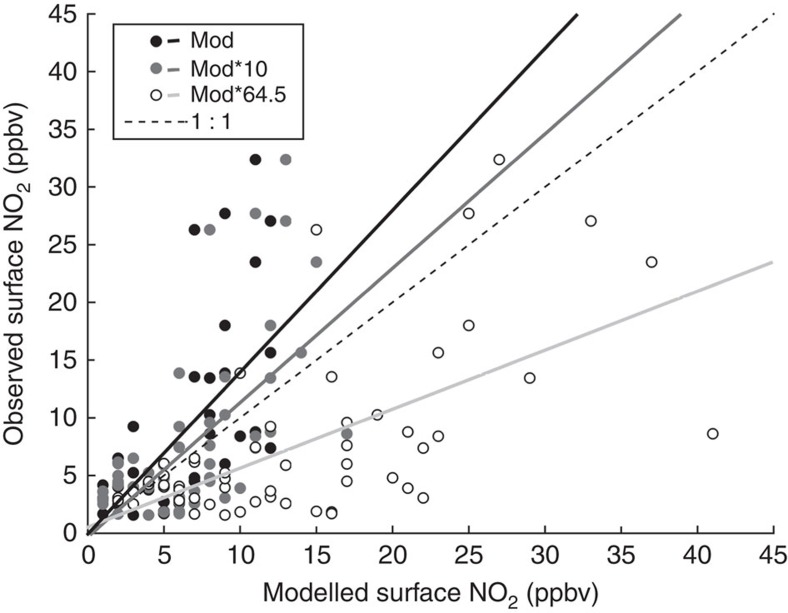
Comparing modelled and measured surface NO_2_ concentrations in El Centro, CA. Surface concentrations of NO_2_ (p.p.b.v.) at an air quality monitoring station in El Centro, CA (CA Air Resources Board; obs) and modelled with WRF-Chem (mod) and WRF-Chem with soil NO_2_ emission rates multiplied by 10 (mod*10) and 64.5 (mod*64.5). All data are from 23 to 29 September 2012. The dashed line represents the 1:1 relationship; all other lines correspond to linear regressions between modelled and observed data (default model *r*^2^=0.44, slope=1.4, intercept=0.14; mod*10 *r*^2^=0.44, slope=1.1, intercept=0.38; mod*64.5 *r*^2^=0.42, slope=0.5, intercept=0.7). All linear regressions were significant at *P*<0.0001.

We then compared modelled surface NO_2_ concentrations in the Imperial Valley with surface NO_2_ measurements collected at an air quality monitoring site (CA Air Resources Board, El Centro-9th Street, CA; Fig. 4). Meteorological conditions were stable, with no rainfall and consistent temperature and radiation during the simulation period (average air temperature=30.29 °C, s.d.=1.9; average daily net radiation=110.9 W m^−2^, s.d.=4.1). WRF-Chem default parameterization performed well, especially on days when NO_2_ surface concentrations were low (for example, day of year (DOY) 269; Fig. 4) and tended to underestimate peak NO_2_ concentrations early in the morning when the daytime boundary layer was developing and pollutants, including NO_2_, were not well mixed (for example, DOY 271; Fig. 4; Supplementary Fig. 1). All model simulations explained significant amounts of variation in observed surface NO_2_ during the simulation period (Fig. 5), with the least amount of bias occurring when soil NO_x_ emissions in the model were increased by an order of magnitude (root mean s.e. (r.m.s.e.)=6.1, 5.7 9.5 p.p.b.v. for model default, model*10 model*64.5, respectively). These results suggest that an improved soil NO_x_ emission factor in WRF-Chem would be close to 10 or 20 × higher than default to match observed surface concentrations of NO_2_ in the Imperial Valley. When soil NO_x_ emissions were increased 64.5 × , the model overestimated surface NO_2_ by 81% across the simulation period. An additional analysis was conducted using only data at local time 13:00–16:00 when the boundary layer was well developed at an average of 1,587 m (s.d.=796 m; Supplementary Fig. 1) and overlapping with Ozone-monitoring instrument (OMI) overpass time (12:00–13:30). However, the results were similar to the analysis including all data (Fig. 5) and therefore are not shown.

While the discrepancies between measured and modelled surface NO_2_ concentrations may be due to the model underestimating soil NO_x_ emissions from local agricultural fields, they may also be due to mischaracterized local meteorology or underestimated NO_x_ emissions inventories from local biomass burning and/or fossil fuel combustion. To investigate alternative sources of error, we first compared locally measured meteorological variables with simulated meteorological variables and found high agreement between modelled and measured air temperature and wind speed during the simulation period (*r*^2^=0.60, r.m.s.e.=2.3 °C and *r*^2^=0.80, r.m.s.e.=0.58 m s^−1^ for air temperature and daily average wind speed, respectively). We also examined the infrared anomaly (fire) data from MODIS and determined that no large biomass burning events had occurred during 20–29 September 2012. The discrepancies between modelled and measured NO_2_ may also be due to under-represented fossil fuel combustion rates; however, the fossil fuel combustion NO_x_ emission data used in the model are known to overestimate anthropogenic NO_x_ sources by 32% in southern California^30^.

**Figure 6:**
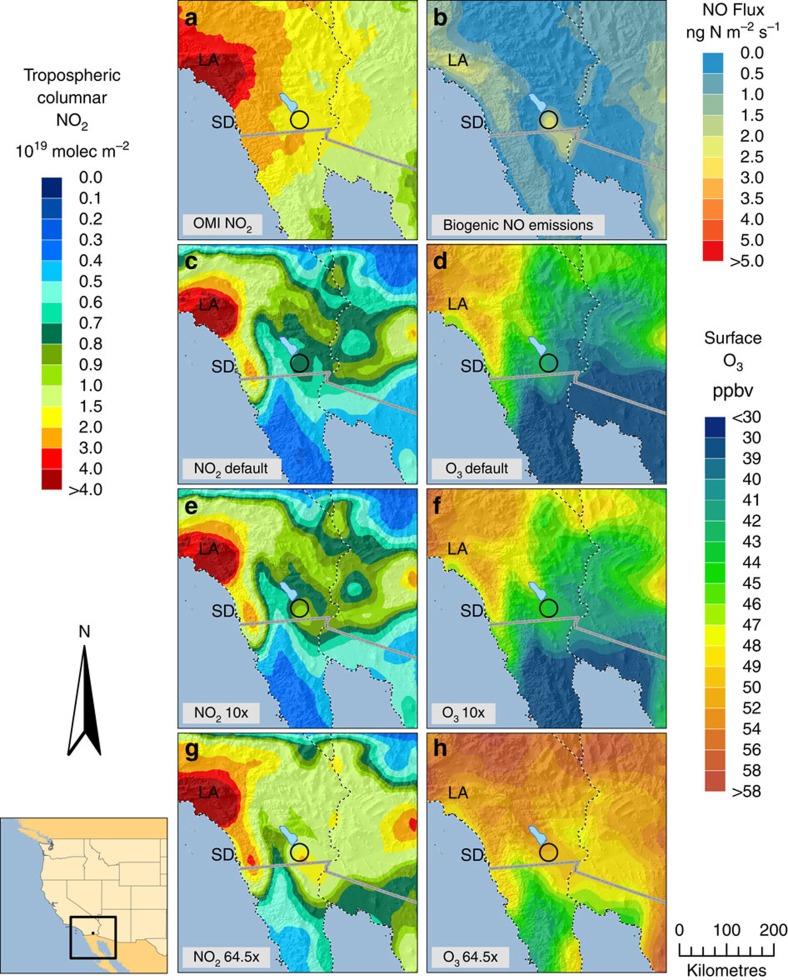
NO_2_ and O_3_ distributions from WRF-Chem and OMI above the Imperial Valley. Distribution of tropospheric columnar NO_2_ retrieved by (**a**) OMI across 3 days (25, 28 and 29 September 2012) measured at 12:00–13:30 local time. WRF-Chem (**b**) surface NO_2_ emissions (ng N m^−2^ s^−1^) and (**c**) tropospheric NO_2_ columns are also shown across the same 3 days at 12:00–13:30 local time. Soil NO_2_ emission rates from cropland were elevated within WRF-Chem (**e**) 10 × and (**g**) 64.5 × above default resulting in higher tropospheric NO_2_ columns. WRF-Chem simulations of surface O_3_ concentrations (p.p.b.v.) are also shown corresponding to the (**d**) default, (**f**) 10 × and (**h**) 64.5 × elevated soil NO_2_ emission runs. All tropospheric NO_2_ column units are in 10^19^ molecules NO_2_ per m^2^. The Imperial Valley study region is circled in black in each panel. Nearby cities are also indicated within each panel as San Diego (SD) and Los Angeles (LA).

We also compared remotely sensed measurements of tropospheric NO_2_ columns with WRF-Chem model simulations. The OMI satellite-derived tropospheric NO_2_ columns suggest that WRF-Chem underestimates NO_2_ above the Imperial Valley by 63% during the simulation period (0.75 and 2.05*10^19^ molecules NO_2_ m^−2^ for WRF-Chem and OMI, respectively; Fig. 6a,c). Elevating soil NO_x_ emissions by an order of magnitude still resulted in the model underestimating observed tropospheric NO_2_ columns by 56% (0.90*10^19^ molecules NO_2_ per m^2^ above the Imperial Valley). However, elevating soil NO_x_ emissions by 64.5 × led to good agreement with observed tropospheric NO_2_ columns (2.0*10^19^ molecules NO_2_ per m^2^ above the Imperial Valley; Fig. 6e,g).

WRF-Chem simulations with elevated soil NO_x_ emission rates resulted in significant increases in surface O_3_ concentration. Under default conditions, WRF-Chem estimates O_3_ at 41.5 p.p.b.v. in the Imperial Valley across 3 days in September 2012 (Fig. 6d). Increasing soil NO_x_ emissions by 10 and 64.5 × increased O_3_ levels by 2.2 and 8.5 p.p.b.v., respectively (Fig. 6f,h). These modelled O_3_ concentrations highlight the sensitivity of air quality to soil NO_x_ emissions in the region and confirm that this air shed is NO_x_ limited.

## Discussion

We find that NO_x_ emission rates from high-temperature agricultural soils in the Imperial Valley are some of the highest ever reported^8^,^31^. Despite differences in fertilizer type and application method, both fertilization experiments resulted in higher than expected NO_x_ fluxes with emission factors ranging between 1.8 and 6.6% over the course of 20–25 days following treatment. Our approximated emission factors are likely an underestimation, as they were derived from non-continuous short monitoring periods highlighting the need for continuous flux measurements. Overall, our results suggest that commonly applied NO_x_ emission factors (typically 1–2% across an entire growing season^5^,^8^,^23^) are highly uncertain and underestimate NO_x_ emissions in high-temperature agricultural systems.

We also find that NO_x_ emissions are best predicted through nonlinear relationships with time since fertilization, soil temperature and soil moisture. Incorporating nonlinear NO_x_ emission responses to these factors into biogeochemical models is becoming more common. In particular, Hudman *et al.*^9^ account for pulse emission responses to fertilization and continuous dependence on soil moisture (instead of distinct wet/dry states) within the Berkeley–Dalhousie Soil NO_x_ Parameterization model, which is available in GEOS-Chem, a global chemistry transport model. However, many models (including the Berkeley–Dalhousie Soil NO_x_ Parameterization model) do not account for NO_x_ emission responses to different levels, chemical species, application methods of fertilization, irrigation events in agricultural systems and nonlinear NO_x_ emission responses to high temperature (>30 °C). Our study highlights these factors as critical functions influencing NO_x_ emissions.

Fertilization management, including the type of fertilizer used and how it is applied, is critical for minimizing the loss of N to the atmosphere in the form of NO_x_. Our fertilization experiment revealed strong nonlinear increases in flux response to increases in fertilizer amount. This extends previous work suggesting that large fertilization events result in higher than expected NO_x_ emissions and splitting fertilization into smaller amounts can greatly reduce NO_x_ emissions^5^. Our experiments also demonstrate that using side-injected granular urea-N-fertilizer results in lower NO_x_ emissions than a dissolved NH_4_NO_3_-N application. Application of 50 kg urea-N ha^−1^ induced an integrated flux that was 30% lower than a 20-kg NH_4_NO_3_-N ha^−1^ treatment. Urea is a more complex N source requiring an extra step before it can serve as a substrate in nitrification and denitrification, while ammonium nitrate is a direct substrate for both nitrification and denitrification. This supports previous research conducted in lower-temperature environments, suggesting that side injection and complex N (for example, urea) limit N trace gas emissions^5^,^32^. Fertilizer management is therefore a critical factor for minimizing N losses to the atmosphere and reducing adverse effects to air quality in high-temperature agroecosystems.

We find NO_x_ emission pulses in response to irrigation alone, a response consistent with a previously described hypothesis that fertilized soil will continue to exhibit pulse NO_x_ emission behaviour with multiple irrigation events^2^. Long-term effects of fertilization on soil NO_x_ production may therefore significantly contribute to annual NO_x_ budgets. Models often assume agricultural systems maintain constant soil-moisture conditions^33^; however, soil surface drying between irrigation events is common in high-temperature arid agroecosystems^34^. Our results stress the importance of understanding how combined fertilization and irrigation practices influence soil NO_x_ emissions.

Soil NO_x_ models often assume NO_x_ emission exponentially increases with temperature until a plateau is reached above 30 °C (ref. 9). Our results highlight nonlinear responses in NO_x_ emissions above 30 °C, where soil NO_x_ emissions increase 38% on average as soil temperatures increase from 30–35 to 35–40 °C (Fig. 1). While exponential relationships between soil temperature and soil NO_x_ emissions are valuable for predicting flux, these responses need to be parameterized to different environmental conditions. This is especially true in high-temperature environments such as the Imperial Valley where microbial acclimation to high temperature and/or increased contributions of deeper and cooler soil layers (>10 cm) to surface NO_x_ emissions may be significant. While we did not observe inhibition of NO_x_ emissions above 35 °C, higher temperatures than those covered in this study (>40 °C) may reveal inhibition of NO_x_ emissions.

We find evidence that the regional air chemistry model WRF-Chem underestimates soil NO_x_ emissions, tropospheric NO_2_ columns and, at times, surface NO_2_ concentrations in the Imperial Valley. Default WRF-Chem simulation of soil NO_x_ emissions from agricultural land was on average 2.0 ng N m^−2^ s^−1^ (Fig. 6b), much lower than observed in the Sorghum field (on average 65 ng N m^−2^ s^−1^ across all measurements). Model simulations of tropospheric NO_2_ columns also underestimated observed values. These results agree with previous research showing that satellite-derived (OMI) tropospheric NO_2_ columns are elevated above agricultural land in the Western United States of America, and that these sources are underestimated in current models^23^. However, the model did not consistently underestimate surface NO_2_ (for example, DOY 272; Fig. 4). While increasing soil NO_x_ emission rates by 64.5 × within WRF-Chem led to strong overestimation of surface NO_2_ observations, it led to good agreement with tropospheric NO_2_ column observations. Our results therefore indicate that there is no single emission factor that can be used to accurately simulate both tropospheric NO_2_ columns and NO_2_ observed at the surface. On the basis of field measurements, soil NO_x_ emissions are highly variable depending on fertilization, soil temperature and soil moisture. We therefore advocate for modifying model structure within WRF-Chem to incorporate these NO_x_ emission dynamics, versus simply increasing emission factors. This is an important area for future research.

High soil NO_x_ emissions are likely contributing to high concentrations of tropospheric O_3_ in the Imperial Valley. Elevating soil NO_x_ emission rates within the model to the point where better agreement was achieved with observations led to significantly higher concentrations of simulated surface O_3_. These model simulations suggest that air chemistry in the region is NO_x_ limited and therefore sensitive to soil NO_x_ emissions. Intensive agriculture in the Imperial Valley and associated high soil NO_x_ emissions may therefore be contributing to poor air quality in the region. Soil NO_x_ emissions may also be contributing to the formation of particulate nitrate, another threat to human respiratory health. The management of fertilizers may be a valuable approach for reducing the negative impacts of agriculture on human health in the Imperial Valley.

There are multiple factors that could lead to WRF-Chem underestimating tropospheric NO_2_ columns and, at times, surface NO_2_ concentrations in the Imperial Valley. While we investigated some of these factors (for example, poorly constrained soil NO_x_ emissions, meteorology and biomass burning NO_2_ sources), more intensive evaluation and improved model parameterization will be required to advance predictive skill of NO_2_ and O_3_ dynamics in the region. First, to improve regional scale modelling of agricultural NO_x_ emissions, future studies require NO_x_ flux measurements across all dominant crop types in the Imperial Valley paired with spatially explicit management data, including irrigation and fertilization practices. Second, improved model structure informed by relationships presented in Fig. 1 will be required for predicting soil NO_x_ emission responses to temperature, irrigation and fertilization. Third, biogenic volatile organic compound emissions in high-temperature irrigated environments^35^ and fossil fuel combustion inventories need to be better constrained to more accurately evaluate the significance of soil NO_x_ sources for tropospheric O_3_ production. This is particularly important as previous work has shown that the EPA's NEI-05 emission data significantly overestimate anthropogenic NO_x_ sources in Southern California^30^. Improving WRF-Chem performance will therefore require evaluation of both model structure and model input data with regard to multiple sources of agricultural and anthropogenic organic compounds and NO_x_ in the Imperial Valley.

In summary, we find high N losses in the form of NO_x_ from high-temperature agroecosystems that are not well represented by a current air chemistry model. Managing soil NO_x_ emissions should be considered in future efforts to improve regional air quality in the Imperial Valley, a region that regularly exceeds government O_3_ standards^14^ and suffers from the highest rates of asthma hospitalizations in California^15^. Our results suggest that smaller doses of side-injected dry fertilizers with complex N formulations may help reduce NO_x_ emissions and thereby increase nutrient-use efficiency. There is growing concern over the sustainability of agricultural production in regions of the world that have been experiencing higher temperatures and more frequent heat waves^1^. Results from this study highlight the need for improved understanding of fertilized high-temperature environments, for better representation in air chemistry transport models and for development of sustainable management of agricultural land in a future warmer climate.

## Methods

### Study location and experimental design

All measurements were conducted in experimental agricultural fields located in the low elevation (−18 m ASL) University of California Desert Research and Extension Center (DREC), Holtville, Imperial County, CA (32°N 48′ 42.6″, 115°W 26′ 37.5″) characterized by deep alluvial soils (42% clay, 41% silt and 16% sand) with 2.34% C and 0.13% N, and a pH of 8.3. The field site experienced historically typical air temperatures during the experiments conducted in 2012 and 2013 (ref. 36). Two N-fertilization studies were conducted under cultivation of a forage cultivar of *S. bicolor* (cv. Photoperiod LS; Scott Seed Inc.) in adjacent fields. Seed of *S. bicolor* were planted at 90,000 plants per ha. High-biomass-producing grasses including sorghum, sudangrass and sugarcane are the 4th most common crop type in the Imperial Valley, behind Alfalfa, pasture and vegetables^37^. These high-biomass-producing grasses typically receive a large fertilizer application at planting (100–150 kg N ha^−1^) followed by smaller applications (50 kg N ha^−1^) throughout the growing season, typically following harvests^38^,^39^. Fertilizers are often applied by injecting anhydrous fertilizer into the sides of furrows or by broadcasting fertilizer on the soil surface. The cheapest form of fertilizer is dry urea, but ammonium nitrate and ammonia are also used^40^. The fields were gravity-fed flood irrigated as needed, usually every 10 days or when soil surface volumetric water content fell below 0.10 cm^3^ cm^−3^. Gravity-fed flood irrigation is the most prevalent irrigation practice in the Imperial Valley; 70% of irrigated crops using water from the Colorado River use flood irrigation^41^. Both fields had beds separated by 1.5 m with 20-cm-deep furrows, and have been used for agricultural production at least since the establishment of DREC in 1912. The first experiment was conducted in a large experimental field (5.3 ha) in 2012 and the second in a smaller field (0.4 ha) in 2013.

### Large-field measurements

Seed of *S. bicolor* were planted on 16 February 2012. Urea fertilizer treatments of 90 kg N ha^−1^ were applied with a 3-m-wide fertilizer spreader on 10 February, 18 June and 16 August 2012, totalling to 270 kg N ha^−1^ per year. Pesticides were applied at 2.1 l ha^−1^ on 30 April 2012 (Lorsban insecticide, Dow AgroSciences, Indiana, USA) and herbicides were applied at 0.84 kg ha^−1^ on 27 March 2012 (Maestro, Nufarm Americas Inc., Illinois, USA). Three harvests were conducted in 2012 on 4 June, 14 August and 12 November (corresponding to DOY 156, 227 and 317, respectively). The second and third growth periods were ratoon crops. The field was left fallow in the winter and was re-seeded on 1 April 2013. Sorghum was planted later in the season in 2013 due to late rains. In 2013, only two harvests were conducted on 19 July and 18 September 2013. Fertilizer treatments were applied on 29 May 2013 at 52 kg N ha^−1^ (side dressed urea), 20 June at 66 kg N ha^−1^ (mixture of urea–ammonium nitrate solution (32%) and 8% ammonium nitrate organic solution), and 20 August 2013 as 96 kg N ha^−1^ (urea–ammonium nitrate 32%), totalling to 214 kg N ha^−1^ per year. No pesticides or herbicides were applied in 2013.

We installed 20 soil collars on 20 February 2012, split between the northwest and southeast quadrants. In each quadrant, 10 collars were divided between two rows separated by three furrows. Soil NO_x_ measurements were conducted throughout the growing season to assess general trends in NO_x_ emissions (21 March, 22 May, 27 June, 7 August and 20 August corresponding to DOY 81, 143, 179, 220 and 233, respectively) and again in 2013 (9 July, 30 July, 19 August, 22 August, 28 August and 2 September corresponding to DOY 190, 211, 231, 234, 240 and 245, respectively). Due to difficulties with instrumentation, not all collars were measured on every sampling occasion.

In the first N-fertilization experiment in 2012, 10 experimental collars received an irrigation and fertilizer treatment (dissolved 20 kg ammonium nitrate-N ha^−1^) on 18 September 2012, while 10 control collars received irrigation only (with five collars in the southeast quadrant and five in the northwest quadrant randomly receiving fertilization). Collars were fertilized concurrent with flood irrigation of the entire field. Before the experimental fertilization, the field had not received fertilization for 32 days (at 90 kg urea-N ha^−1^). During this experiment, each collar was measured on 18 September, 21 September, 25 September, 5 October and 13 October (corresponding to DOY 262, 265, 269, 279 and 287, respectively). Plant canopy height next to each collar was also measured on those dates.

### Small-field measurements

A similar N-fertilization experiment was conducted in 2013 in an adjacent Sorghum field (planted on 1 April 2014). We installed soil collars on 23 July 2013 in a randomized block design where the field was split into three blocks each with six rows of control, six rows of low (50 kg urea-N ha^−1^) fertilizer treatment and six rows of high (100 kg urea-N ha^−1^) fertilizer treatment. One soil collar was established within each treatment per block (nine soil collars total with three replicates per treatment). Fertilizer granules (urea) were side-injected into the furrows using a tractor, immediately followed by flood irrigation on 29 July 2013 (DOY 210). Before the experimental fertilization, the crop had not received fertilization. Soils in this field had not been fertilized since the cultivation of a previous crop 5 months before. Measurements were collected on 30 July, 7 August, 13 August and 19 August 2013 (corresponding to DOY 211, 219, 225 and 231, respectively). Plant canopy height next to each collar was also measured on those dates.

### Soil NO_x_ flux measurements

NO_x_ chamber measurements were conducted using the static chamber technique. Soil collars (made of polyvinyl chloride with a diameter of 20 cm and height of 10 cm) were inserted 4–6 cm into the soil on the top of furrows. A custom-built chamber was set on top of soil collars and set into place using a rubber seal. The chamber was also made from polyvinyl chloride with a mixing fan mounted on the inside and reflective tape covering the outside of the chamber^42^. Air was pulled from the top of the chamber at 1 l min^−1^ and routed to a portable NO monitor (Nitric Oxide Monitor Model 410 with NO_2_ converter Model 401, 2B Technologies, Colorado, USA) where depletion of O_3_ is measured using UV absorbance (detection range: 2–2,000 p.p.b.; precision: ±1.5 p.p.b.; measurement rate: 0.1 Hz). This system converts all NO_2_ to NO using a molybdenum converter before sending sample air to the NO monitor, therefore measurements are expressed as NO_x_ flux (NO+NO_2_). This technique is similar to conventional chemiluminescence analyzers; however, we found our system to be better suited to high-emission environments compared with chemiluminescence instruments in preliminary studies conducted in both field and lab settings. Soil NO_x_ flux was calculated using the rate of increase in NO_x_ concentration within the first 3 min of placing the chamber onto the collar. We used linear regression to determine rates of change from an average of nine points in the regression or 1.5 min of data.

### Ancillary soil sampling and inorganic soil N analysis

Each flux measurement was paired with soil temperature, moisture and inorganic N measurements. Soil temperature was measured next to each collar at 2 and 10 cm depth (Fluke 51 II Thermometer (Wilmingtion, NC, USA)). A soil core (1.5 cm diameter) was extracted next to each collar following the NO_x_ measurement (0–10 cm). The core was homogenized in a bag before removing 15 g for measuring soil volumetric water content and 2.5 g for inorganic N extraction at a 1:10 soil weight-to-solution volume ratio using 2 M KCl. Extracts were put on ice until transported back to the lab, where they were processed within 24–48 h. Samples were shaken for 1 h at room temperature, centrifuged and filtered through Whatman no. 40 filter paper (11 μm) and frozen until further processing. Filtrates were acidified and analysed by automated cadmium coil reduction for nitrate/nitrite (Seal Analytical Inc., AQ2 Discrete Analyzer (Mequon, Wisconsin). NO_3_ and NH_4_ are expressed in μg N g^−1^ dry soil.

### Statistical analyses

In a comprehensive analysis of all observed NO_x_ fluxes, nonlinear generalized additive modelling was used to assess the influence of environmental parameters on NO_x_ flux via the GAM function in R (v.3.1.1, Vienna, Austria). The model included explanatory variables NO_3_, NH_4_, soil temperature (averaged between 2 and 10 cm depth), soil volumetric water content (averaged across 0–10 cm depth) and days since fertilization. The best-fitting parsimonious model was selected using Akaike's information criterion. For visualization, NO_x_ fluxes were binned according to environmental variables (days since fertilization, soil volumetric water content and soil temperature). Third-order polynomials were fitted to the relationships between binned average NO_x_ flux and soil volumetric water content and soil temperature. A Gaussian equation was fitted to the relationship between binned average NO_x_ flux and days since fertilization. All fitting was performed in Matlab (v.R2014a, The Mathworks, Inc. USA). Repeated measures analyses of variance were conducted independently on each N-fertilization experiment to test for significant effects of treatment (fertilized or control) on NO_x_ emissions. Pairwise comparisons using the Bonferroni adjustment were conducted to explore differences between treatments at specific time points. NO_x_ emissions were log transformed to meet homogeneity of variance assumptions. Analysis of variance and Bonferroni tests were performed in R (v.3.1.1, Vienna, Austria). To estimate total N released as NO_x_ in response to treatments, we performed numerical integration via the trapezoidal method in Matlab. These integrated values are only approximate and are most likely an underestimate of total flux, as peak fluxes may not have been captured with discontinuous measurement techniques.

### Regional air quality modelling

We evaluated the influence of soil NO_x_ emissions on regional air quality using the WRF-Chem (version 2.0). The WRF-Chem model^29^,^43^ is a regional air quality model that can be used for weather forecasting and simulating gas-phase chemistry, including NO_x_ and ozone chemistry at an hourly time step. With its nested grid capability, WRF-Chem-simulated quantities can be more easily compared with a wide range of *in situ* and remote sensing data collected at different temporal and spatial resolutions. A nested grid configuration was used in this study with the centre in the Imperial Valley, CA. The resolution of fine grid was 12 × 12 km and the outer domain was 36 × 36 km. Table 1 lists the model configuration options employed in this study.

The NARR (North American Regional Reanalysis) data at 0000, 0600, 1200 and 1800 UTC were used for initializing and specifying the temporally evolving lateral boundary conditions. The US National Emissions Inventory emissions data (NEI-05; version 2) was used in the simulation as background emission (US Environmental Protection Agency, 2010). The NEI-05 data are likely a high estimate for anthropogenic NO_x_ sources in the Imperial Valley. Previous work in Los Angeles County has shown that anthropogenic NO_x_ sources in NEI-05 are overestimated by 32% (ref. 30). The land-use data used in this study is the US Geological Survey land-use data. Biogenic emissions of volatile organic compounds and soil NO_x_ emissions are calculated using the Model of Emissions of Gases and Aerosols from Nature (MEGAN v2.0 (refs 44, 45)). In MEGAN, gridded emission factors are based on global data sets of four functional plant types (broadleaf trees, needle-leaf tree, shrubs/bushes and herbs/crops/grasses), where the herbs/crops/grass category has a higher emission factor compared with the other plant types^46^. In this version of MEGAN, soil N (NO, NO_2_ and NH_3_) emissions are a function of temperature only; production and loss of NO_x_ within the canopy is not considered. Previously reported canopy uptake rates are low, ranging up to 3 ng N-NO_2_ m^−2^ s^−1^ under high light and high NO_2_ concentrations (NO_2_=5 p.p.b.)^47^. However, canopy uptake rates in high-emission and high-temperature environments are uncertain and require further research^36^,^47^,^48^,^49^,^50^. Pulse NO_x_ emissions following fertilization events are also not considered in the model. The emission factor for agricultural soils is 6 ng N m^−2^ s^−1^ at a standard temperature of 273.15 K (ref. 46). Due to assumptions made in the satellite observations, we apply an averaging kernel to the WRF-Chem simulations to allow comparison with the space-borne OMI (described below)^51^,^52^.

### Modifications to a regional air quality model

To evaluate the sensitivity of air quality to soil NO_2_ sources in the Imperial Valley, we modified the strength of soil NO_x_ emissions from irrigated agricultural land. Other land types such as surrounding urban and dry native lands were not manipulated. We elevated WRF-Chem emission rates by a factor of 10 and 64.5, resulting in simulated soil NO_x_ emissions in Imperial Valley croplands near 20 and 129 ng N m^−2^ s^−1^, which are representative of the range in mean and median flux values collected under both average and recently fertilized conditions in the field. It is important to note that this modelling exercise simply increases emission rates and does not account for the observed nonlinear pulse NO_x_ emission events that occur in response to fertilization.

All simulations were run for 7 days in September 2012 (23–29 September 2012), with several days as spin-up time. These simulations were compared with measurements of surface and tropospheric NO_2_ columns above the Imperial Valley.

### Comparing modelled and measured NO_2_

Evaluation of WRF-Chem model performance was assessed through comparisons with surface and satellite observations. First, we compared modelled with measured surface NO_2_ in the Imperial Valley. Surface NO_2_ concentrations are measured by the California Air Resources Board at an air quality-monitoring site located 11.3 km west of DREC on 9th Street, El Centro, CA (latitude: 32.79222; longitude: −115.563). This site is not near a point source and provides representative concentrations of pollutants for the Imperial Valley. Surface NO_2_ measurements are made by first reducing all NO_2_ to NO using heated molybdenum surfaces and then measuring the chemiluminescent reaction of NO with O_3_ (ref. 53). Comparisons between modelled and measured surface NO_2_ concentration were made for all WRF-Chem model simulations (default, 10 × and 64.5 × elevated soil NO_x_ emission). WRF-Chem model performance was assessed using linear regression and the coefficient of determination (*r*^2^). Model bias was estimated using the absolute r.m.s.e. between modelled and observed surface NO_2_ concentrations.

To evaluate the model's ability to simulate local meteorology, we compared daily average wind speed (m s^−1^) and air temperature (°C) measured at the El Centro air quality monitoring station and simulated by the model. Model performance was assessed using the coefficient of determination (*r*^2^) and the absolute r.m.s.e.. We evaluated local sources of NO_x_ from biomass-burning events using MODIS images. MODIS images are publically available and were assessed for 20–29 September 2012. We also analysed meteorological data from a weather station located at DREC (managed by the California Irrigation Management Information System, www.cimis.water.ca.gov) to investigate how rainfall (mm), air temperature (°C) and net radiation (W m^−2^) changed during the simulation period.

We also assess WRF-Chem performance using remotely sensed tropospheric columnar NO_2_ by OMI on board the Aura satellite. OMI measures radiation in the broad visible spectrum between 264 and 504 nm^54^. OMI has near daily contiguous global coverage with moderate spatial resolution (60 cross-track ground pixels ranging from 13 × 24 to 128 × 40 km at the edge of a sampling swath). We use the level 2 (version 2.0) KNMI-DOMINO product (Royal Netherlands Meteorological Institute). The KNMI product and its errors are described in detail by Irie *et al.*^55^ and Boersma *et al.*^56^,^57^. Briefly, the KNMI-DOMINO product determines the stratospheric portion of the column by assimilating slant columns in the TM4 chemistry transport model, with an uncertainty in the stratospheric NO_2_ column of near 0.3*10^15^ molecules per cm^2^ (ref. 58). The tropospheric air mass factor is determined using the formulation of Palmer *et al.*^59^ and Boersma *et al.*^60^ to convert slant columns to vertical columns^57^. The KNMI product is known to compare well with aircraft measurements of NO_2_ in urban regions (*r*^2^=0.67, slope=0.99±0.17)^61^, while tending to overestimate NO_2_ in remote regions^62^,^63^. When compared with ground-based measurements in China, the biases of the KNMI product were less than 10% (ref. 55). The KNMI product is also known to have less systematic seasonal error compared with the NASA product^52^. Due to satellite data having irregular grid boxes, we re-gridded to regular grid boxes at a 0.4° resolution using area-weighted average for illustrative purposes (Fig. 6). During the WRF-Chem simulation period, an average of eight OMI pixels fell within the Imperial Valley region. We present OMI data averaged across 25, 28 and 29 September 2012, as these were all days during the WRF-Chem simulation period when OMI data were available, the cloud radiance fraction was <50% and the target region was not on the edge of the swath (excluding pixels with a viewing angle >45°).

## Additional information

**How to cite this article:** Oikawa, P. Y. *et al.* Unusually high soil nitrogen oxide emissions influence air quality in a high-temperature agricultural region. *Nat. Commun.* 6:8753 doi: 10.1038/ncomms9753 (2015).

## Supplementary Material

Supplementary Information Supplementary Figure 1

## Figures and Tables

**Table 1:** WRF-Chem configuration.

**Atmospheric process/inputs**	**Model option**
Surface layer	MM5
Land surface	Noah^64^
Boundary layer	YSU^65^
Cumulus clouds	G3 (ref. 66)
Cloud microphysics	Lin^67^
Gas-phase chemistry	RADM2 (ref. 68)
Aerosol chemistry	MADE^69^/SORGAM^70^
Horizontal resolution	36 km For the outer domain, 12 km for the inner domain
Vertical layers	27

WRF-Chem, Weather Research and Forecasting with Chemistry model.
