# Supplementary material for: Unusually high soil nitrogen oxide emissions influence air quality in a high-temperature agricultural region
Source: Nat Commun. 2015 Nov 10;6:8753. doi: 10.1038/ncomms9753 (PMC4659929; doi:10.1038/ncomms9753)
Supplement: Supplementary Information — Supplementary Figure 1 [file ncomms9753-s1.pdf]

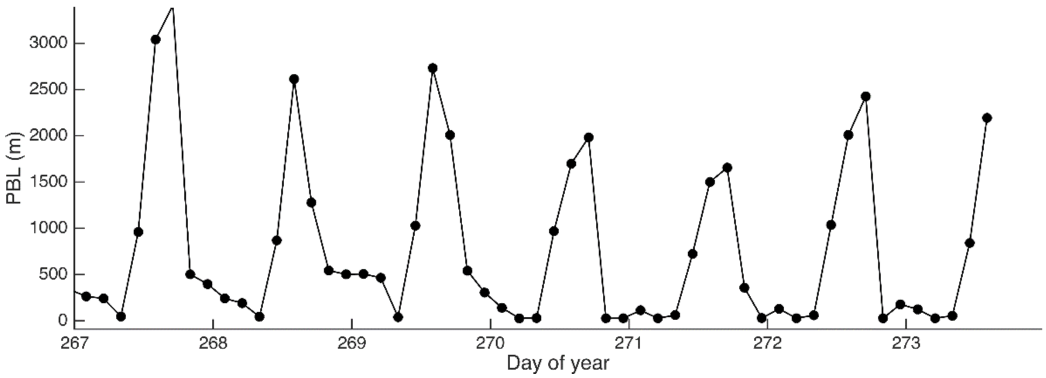

Supplementary Figure 1. Planetary boundary layer height over time. Planetary boundary layer height (PBL, m) simulated by WRF-Chem above the Imperial Valley, CA during September 23-29, 2012.
